# Supplementary material for: The genome sequence of the Brown garden snail, Cornu aspersum (O.F.Müller, 1774) (Stylommatophora: Helicidae)
Source: Wellcome Open Res. 2025 Sep 8;10:487. [Version 1] doi: 10.12688/wellcomeopenres.24859.1 (PMC12616119; doi:10.12688/wellcomeopenres.24859.1)
Supplement: Peer review history

# Reviewer report: Reviewer response for version 1

**DOI:** 10.21956/wellcomeopenres.27383.r140155

**Published:** 2025-12-26

**Authors:** Deborah Power

**Affiliations:** 1 Campus de Gambelas, Centro de Ciencias do Mar, Universidade do Algarve, Algarve, Portugal

I enjoyed reading this MS as it is clearly written and concise. The objective is clear and so my evaluation is primarily linked to the fundamental aim, which is to generate high quality genomes and provide open acess to the data and the means of their generation. As such I consider that overall this has been acheived particulalry since much of the methodology of the study complies with the protocols.io developed as part of the "Tree of life" exercise. However, there were some aspects, related to parts of the methods that I feel could be improved to make the technical approach totally transparent, and reproducible. I make some observations about additional information that would be useful to have in the Introduction and then some small comments related to parts of the method description. I briefly identify my main suggestions/comments below:

 1. In the introduction it would have been helpful to have an indication that the *Cornu aspersum *is diploid.

 2. It is indicated that *C. aspersum* is hemaphrodite but it would have been useful to indicate that they undergo sexual reproduction generally. 

 3. Linked to the previous point I found the comment that they have an "adaptable reproductive strategy" without real meaning, and perhaps here a short but specific statement about their reproductive strategy could be provided.

 4. I had a question about if/how using specimens from different geographical locations for the genome and transcriptomes influenced the results.

 5. I guess the mitochondrial DNA was only obtained for the specimen from Luton. That might have given some insight into the effect of geography or perhaps this would have been found if the transcriptome was compared to the genome.

 6. In the section about seqeuncing, I could not find reference to protocols.io for the sequencing approach and so they seem very vague compared to the more precise methodology reports of the other sections. Hi-C I would have apprecated a it more definition in relation to the reagents e.g., Arima Genomics, but country not mentioned, Diagnocine Power Masher-II (no fabricant mentioned), Qubit HS Assay Kit  - is this the same as the Qubit dsDNA high sensitivity assay??, its not clear as written, Covaris E220 sonicator is from where and what were the setting used?

 I could continue in relation to the library preparation, but I hope the points I raise indicate what I think is missing in general from this section, which as I said does not seem to be covered by protocols.io and so makes this section of the MS much less clear.

 Assembly methods are thorough and comprehensible.

 One comment about the Genome sequence report. I suggest a change to the following statement, I find it ambiguous and imprecise:

 These chromosome-level scaffolds, confirmed by Hi-C data, are named according to size....

 This I think would be clearer if written as follows: 

 These chromosome-level scaffolds, confirmed by Hi-C data, are sequentially numbered from largest to smallest size....

 In Table 1, why is "other somatic animal tissue" indicated under tissue. This is ambiguous particulalry since there virtually no description about what tissues were harvested and how. Similarly, it is indicated anterior body was used for the genome sequencing but not explained anywhere in the methods how this is identified anatomically.

Are sufficient details of methods and materials provided to allow replication by others?

Partly

Is the rationale for creating the dataset(s) clearly described?

Yes

Are the datasets clearly presented in a useable and accessible format?

Yes

Are the protocols appropriate and is the work technically sound?

Yes

Reviewer Expertise:

Comparative Endocrinology, Comparative Genomics, Fish, Bivalves, Invertebrates, Biotechology

I confirm that I have read this submission and believe that I have an appropriate level of expertise to confirm that it is of an acceptable scientific standard, however I have significant reservations, as outlined above.

# Reviewer report: Reviewer response for version 1

**DOI:** 10.21956/wellcomeopenres.27383.r134586

**Published:** 2025-11-13

**Authors:** Maitrya Sil

**Affiliations:** 1 Jain University, Bengaluru, Karnataka, India

This is a well written manuscript. The quality of the assembly looks good. The genome completeness is a little low. The authors should provide reasons behind the low completeness. I suggest using the **most recent version of BUSCO** to alleviate this issue. The accession number of the mitogenome is also missing. If has been submitted separately, I suggest that the authors provide the accession number.

Are sufficient details of methods and materials provided to allow replication by others?

Yes

Is the rationale for creating the dataset(s) clearly described?

Yes

Are the datasets clearly presented in a useable and accessible format?

Yes

Are the protocols appropriate and is the work technically sound?

Yes

Reviewer Expertise:

NA

I confirm that I have read this submission and believe that I have an appropriate level of expertise to confirm that it is of an acceptable scientific standard, however I have significant reservations, as outlined above.

# Reviewer report: Reviewer response for version 1

**DOI:** 10.21956/wellcomeopenres.27383.r134584

**Published:** 2025-10-13

**Authors:** Peri Bolton

**Affiliations:** 1 The Ohio State University Department of Evolution Ecology and Organismal Biology, Columbus, Ohio, USA

Generally good standard long-read genome assembly paper, just some extra thoughts:

 The paragraph on the distribution feels a little bit repetitive in discussion of coastal distribution. 

 One thing I am not sure I understand: The GC content on the snail plot is basically completely uniform across the genome. I was under the impression that GC content varied across the genome, so I do not understand why this plot does not reflect this. However, I see that many other assemblies feature this, so I am not sure that this is exactly a red flag. 

 This is in response to my "Partly": Not sure why the genome annotation wasn't presented in this manuscript, given that RNAseq was conducted and reported. The authors suggest that it will be done in the future. I do not know if this is standard practice for these genome reports. I have found examples of others that do include annotation reports.

Are sufficient details of methods and materials provided to allow replication by others?

Yes

Is the rationale for creating the dataset(s) clearly described?

Yes

Are the datasets clearly presented in a useable and accessible format?

Partly

Are the protocols appropriate and is the work technically sound?

Yes

Reviewer Expertise:

Conservation genomics

I confirm that I have read this submission and believe that I have an appropriate level of expertise to confirm that it is of an acceptable scientific standard, however I have significant reservations, as outlined above.

# Reviewer report: Reviewer response for version 1

**DOI:** 10.21956/wellcomeopenres.27383.r134588

**Published:** 2025-10-09

**Authors:** Dongxiu Xue

**Affiliations:** 1 Qingdao Marine Science and Technology Center, Qingdao, China

This manuscript presents a high-quality, chromosome-level genome assembly of *Cornu aspersum* generated under the Darwin Tree of Life Project. The use of PacBio HiFi and Hi-C data has produced a robust assembly and provides an important reference for terrestrial gastropods. The detailed documentation of protocols and data accessibility is exemplary.

 however, the authors should clarify whether the individual used for PacBio HiFi sequencing is of the same sex as those used for transcriptome annotation and Hi-C scaffolding. If the sex of each specimen is known, it would be useful to state this explicitly in the text, as potential sex-linked genomic differences or sex-biased expression may influence assembly structure and annotation outcomes.

Are sufficient details of methods and materials provided to allow replication by others?

Yes

Is the rationale for creating the dataset(s) clearly described?

Yes

Are the datasets clearly presented in a useable and accessible format?

Yes

Are the protocols appropriate and is the work technically sound?

Yes

Reviewer Expertise:

Evolutionary biology, genomics, genome evolution, population genomics

I confirm that I have read this submission and believe that I have an appropriate level of expertise to confirm that it is of an acceptable scientific standard.
